# The American Dental Association

**Published:** 1877-09

**Authors:** 


### AMERICAN JOURNAL OF DENTAL SCIENCE.

**Vol. XI. THIRD SERIES—SEPTEMBER, 1877. No. 5.**

### ARTICLE I.

### *American Dental Association.*

The Seventeenth Annual Session of this Association was held in Chicago, August 7th, 8th, 9th and 10th, 1877, at the Grand Pacific Hotel; Dr. George W. Keely, of Ohio, presiding. Among those present were the following members of the profession:

George R. Thomas, George L. Field, Joseph Lathrop, H. H. Jackson, Detroit; C. D. Cook, A. H. Brockway, Brooklyn; J. Taft, W. D. Howe, E. Osmond, H. A. Smith, D. W. Clancey, Cincinnati; W. O. Kulp, Davenport, Ia.; Homer Judd, W. N. Morrison, C. W. Spaulding, St. Louis; Charles P. Prayne, C. R. E. Koch, Gorton Nichols, T. W. Brophy, E. S. Talbot, George H. Cushing, J. N. Crome, A. W. Harlan, M. S. Dean, E. Housinger, E. D. Swain, W. C. Dwyer, W. W. Allport, A. W. Freeman, Chicago; B. M. Wilkerson, Baltimore; L. D. Shepard, George F. Waters, Jacob L. Williams, Boston; J. F. Morrison, Ottawa, Ill.; Thomas Fillebrown, Portland, Me.; M. H.

Webb, Lancaster, Pa.; M. S. Hand, Joliet, Ill.; K. B. Davis, Springfield, Ill.; W. H Morgan, J. C. Noel, Nashville, Tenn.; E. C. Stone, Galesburg, Ill.; Corydon Palmer, F. M. Odell, W. H. Atkinson, John Allen, New York; C. A. Ketcher, Rockford, Ill.; J. A. Watling, University of Michigan, Ann Arbor; L. C. Ingersoll, Keokuk, Iowa; J. A. Harris, Pontiac, Mich.; C. S. Smith, Elgin, Ill.; John Campbell, Bloomington, Ill.; William H. Goddard, Louisville; J. H. McQuillen, G. T. Barker, Philadelphia; F. H. Rehwinkle, Chillicothe, O.; George J. Fredericks, New Orleans; Horace A. Mansfield, Evanston, Ill.; J. C. McCoy, Booneville, Mo.; George W. Keely, Charles J. Keely, Oxford, O.; S. M. Sturges, Quincy, Ill.; S. B. Brown, Ft. Wayne, Ind.; Merrit Wells, Indianapolis, Ind.; Edgar Palmer, LaCrosse, Wis.; J. M. Hurtt, Peoria, Ill.; A. S. Chapman, Princeton, N. J.; A. O. Rawls, J. B. Kidd, Lexington, Ky.; S. B. McDonald, Conneautsville, Pa.; J. P. Wilson, Burlington, Ia.

Reports.—The local Committee of Arrangements reported on the accommodations secured for the members; the Executive Committee suggested a change or two in the programme; the Committee on Credentials made its report of those who were accredited delegates; and the Publication Committee reported on the publication and distribution of the minutes of the last session, apologizing for their late appearance and charging up the delay to the stenographer. The Reports were adopted.

Dr. Atkinson, Chairman of the Special Committee, presented a report recommending the division of the Association into permanent sections, similar to those in the American Medical Association. The report was made the special order for Thursday morning at 10 o'clock.

Dr. M. S. Dean, of Chicago, Chairman of the Committee on Physiology, read an exhaustive paper on the subject of Vital Action. The gentleman showed, by a long course of argument, that vital action resists the progress of caries in the dentine, and that under certain circumstances changes occur within the structure of the dentine from irritation produced by caries, which are due to vital action.

Dr. Atkinson disputed the propriety of the distinction made between organic and inorganic action, claiming that it was all vital action, and all operated by one power.

Dr. Chase, of St. Louis, and Dr. Waters, of Boston, cited cases in support of the theory of vital action.

Dr. Taft moved that the privileges of the floor be granted to Dr. Robertson, of Manchester, N. H., an old member of the profession, who had had the misfortune to lose his sight. The motion was carried, and Dr. Robertson cited his own case to show that dentine resists decay and forms new deposits.

Dr. Barker, of Philadelphia, took the same ground as that taken by Dr. Atkinson. Vital action and chemical action were the same, differing only in degree.

Dr. Judd, of St. Louis, claimed that the deposit of earthly matter in the cells was brought about by vital action. In his opinion, there was quite a difference between vital and chemical action, and in many cases the former had a great influence in determining the latter.

The discussion was prolonged until 1 o'clock, when the session adjourned till the afternoon.

AFTERNOON.

The afternoon session began at 3 o'clock. The Secretary said he had a report written by Dr. J. N. Farrar, of Brooklyn, N. Y., of the Committee on Physiology, on the subject of "North Light vs. Sunlight," but that, while the chirography was plain enough, the entire absence of anything like punctuation marks, and the peculiarity of the wording, made it impossible to read the report in such a way as to do justice to the author The latter had expressed in his letter to the Secretary a fear that the reading of the paper would not do him justice, and, judging from the progress which he (the Secretary) had made in deciphering it, he was of opinion that this fear was well grounded.

The reading of the paper was deferred until Wednesday morning.

Dr. L. C. Ingersoll, of Keokuk, Ia., read a paper entitled, "Is the Dental Pulp Essential to the Integrity of the Dental Structure?" The doctor's answer to his own question was, in brief, that too much stress had been laid on saving the pulp, and there had been too long-continued efforts to save its vitality. In his opinion it was no longer a question of the preservation of the pulp, but the preservation of the teeth. He advised the removal of the devitalized tissue, and in case this was impossible, what remained should be dried of its watery portion and embalmed. Above all, he would advise trying to save the teeth rather than the pulp.

Dr. A. O. Rawls, of Lexington, Ky., and Dr. Barker, of Philadelphia, took precisely opposite views, and the latter stated, incidentally, that Philadelphia dentists were not much of a success in capping pulps.

Dr. Sturges, of Quincy, on the other hand, was disposed to defend the paper. In Keokuk it would be considered a very practical paper, and it would be thought a practical paper in Philadelphia if they only knew something there. [Laughter.]

Dr. Robertson drew on his own experience in connection with this matter.

Dr. Ingersoll made another plea for the salvation of the tooth rather than the pulp.

The discussion was long drawn out,—Drs. Robertson, Allen, Atkinson, and others contributing to the general information on the subject.

Dr. F. H. Rehwinkle read a paper on Pyorrhea Alveolaris.

After some miscellaneous business of little importance the Association adjourned until Wednesday morning.

WEDNESDAY, AUGUST 8TH, 1877.

The Treasurer, Mr. William H. Goddard, presented his annual report, from which it appears that the balance on hand Aug. 1, 1875, was $175; cash collections during the year, $715; total, $890; expenditures, $601.36; balance to date, $288.64.

Dr. Farrar's paper on "*North Light vs. Sunlight*" was then read by the Secretary. It covered some fifty pages of legal cap, and the reading consumed the better part of an hour. The paper related chiefly to the health of dentists, the author holding that steady attention to work in a north light was productive of weakness and disease. Health and comfort suggested the freer use of sunlight, which, according to Sir David Brewster, was the very life-blood of nature. The vital statistics of hospitals would reveal the fact that those who enjoyed the sunlight thrived far better than those who did not. And yet some physicians were so blind to these facts that they persisted in the worship of that dangerous idol, north light. The detrimental effect growing out of the absence of sunlight was seen among the operatives of our factories, among the poor huddled together in dark tenement houses, in their pale, lean, cadaverous faces, and the general air of weakness which seemed to be one of the accompaniments of their existence. The author of the report detailed various experiments made with bay-windows placed in such positions as to get the greatest share of sunlight, and recommended that, where it was possible, dentists should have a southwest bay-window; through which the warm, health-giving rays of the sun could shine from morning till night.

Dr. McQuillen, of Philadelphia, said he used to be in favor of north light. That was when he was young. At that time a medical friend told him he would change his views when he reached more mature years. That change had come. He would even advise people to walk on the sunny side and not the shady side of the street, although he was compelled to admit that it might be well in July and August to look out for sunstrokes.

Dr. Spalding, of St. Louis, thought a liberal eating of fruit would prove of advantage to those who could not get the sunlight.

Dr. Waters, of Boston, related how he had succeeded through the agency of the sunlight, in hastening the growth of the wriggling tadpole into the juvenile frog. He, also, was in favor of the fruit diet.

Dr. Rehwinkle, of Chillicothe, O., could not speak too highly of the paper. It should be published and studied. Mothers should read it and follow out its suggestions. He alluded to the prevalent American practice of shutting up a house so as to keep out the sunlight during the day, and remarked that it was all wrong.

Dr. Thomas, of Detroit, said a south room was to be preferred even on the score of comfort, as it was cooler than a north room. As far as the light was concerned, there was no question that the light coming from the south was superior to that from the north.

Dr. Barker, who started out with the rather incredible statement that he was a modest man, undertook to overthrow the conclusions arrived at in the paper, which were the obvious sense of the Association. He cited his own case, said he had operated for twenty years in a north light, alluded to the ways of artists and mechanics who used the clear north light, and concluded by showing a sun-burned hand as an evidence that the north light had exercised no detrimental effect upon him.

Dr. Freeman, of Chicago, said it was not the fault of some people that they were strong and hearty enough to eat sole leather. [Laughter.] In like manner, Dr. Barker's strong, vigorous nature could withstand the baneful effects of the north light.

Dr. Atkinson was disposed to criticise the paper. In the first place, light was light, whether it came from the north or from the south, and he believed that more eyes had been injured by too much than by too little light. There was too much of an easy-going disposition to accept the statements of those who had made experiments in this matter as conclusive, but authorities could not, in his opinion, sway the eternal principles of verity.

The discussion proved very fruitful of "opinions," and the subject was finally dropped with the implied recommendation that dentists should take frequent sun-baths, even if they did not draw, plug, and fill people's molars and incisors by the aid of the sunlight.

At this point the Association adjourned until 3 p. m. to enable the members to accept an invitation to visit the Board of Trade and learn how young operators get their eye-teeth cut in that modern Babel.

On re-assembling, Dr. Rehwinkel moved to admit as honorary members Dr. Adolf Petermann, of Frankfort-on-Main, and Dr. George Von Langsdorff, of the University of Freiberg. The motion was carried, and the Secretary instructed to notify these gentlemen of their election.

Some discussion took place on Dr. Rehwinkel's paper on "*Pyorrhea Alveolaris*," i. e., loosening of the gums. The paper itself took the ground that there was a cure for this disease, which had previously been considered incurable, and the mode of treatment which it recommended was the continuous removal of the tartar and the treatment of the gums with thymol in alcohol.

Dr. Rawls, of Lexington, Ky., did not think the author of the paper had touched the real cause of the disease. In fact, according to Dr. Rawl, the cause of the disease was not known, and the proposed treatment would not, in his opinion, meet the case.

Dr. Shepard, of Boston, spoke favorably of the treatment pursued by Dr. Riggs, of Connecticut. According to Dr. Riggs, this disease was constitutional in its origin and results, and his treatment consisted in the surgical removal of the deposit on the teeth. Dr. Shepard cited a case where a patient had lost his appetite, suffered from dyspepsia and other uncomfortable symptoms, and this treatment of Dr. Riggs' had restored him to perfect health in six weeks. In Dr. Shepard's opinion, surgical treatment alone insured recovery.

Dr. Palmer, of New York, thought too much time was being spent on this subject. In his opinion this disease had received more attention and had been raised to more importance than it deserved. Any dentist could treat it by following the ordinary teachings of his profession.

Dr. Barker, of Philadelphia, contended for a treatment which should take into account systemic remedies and hygienic rules.

Dr. Atkinson was disposed to quarrel with those of his brethren who, in the course of the discussion, had called this disease an abscess. It wasn't an abscess, or any such thing, but an ulcer. Dr. Atkinson put no faith in mere surgical treatment, and recommended, in addition to the cleaning of the tooth and the scraping away of the deposit, the washing of the tooth with warm salt and water, after which the pocket should be filled with aromatic sulphuric acid. The Doctor also paid considerable attention to the subject of diet, the proper care of the teeth, etc.

Dr. McQuillen, Dr. Rehwinkel, Dr. Judd, Dr. McDonald, and others prolonged the discussion until after 6 o'clock, when an adjournment was had until the evening session.

### Evening Session.

The Association was called to order at 8:15 o'clock.

Dr. Allen, of New York, introduced a resolution providing for the appointment of a committee of three to revise the code of dental ethics so that a harmony might be created that did not now exist.

The resolution was discussed pro and con by Drs. Spaulding, of St. Louis, Morgan, of Nashville, Tenn., and Allport, of this city, and was finally indefinitely postponed.

Dr. Edgar Palmer, of LaCrosse, Wis., then read a paper entitled "Alcohol, and Its Effect upon the Teeth." The paper was full of interest and lengthy. It first treated on the effect of alcohol on the physical system, wherein it was argued that alcohol was without nourishment, and the taking of it into the stomach was to superadd to the necessities of organic life. Secondly, it treated of its effect on the membranes, and the conclusion was reached that alcohol contributed largely to the degeneracy of the dental organs.

The paper was dicussed by Dr. Atkinson, of New York, and placed on file and ordered printed.

Committee reports were next called for, but, no reports being ready, they were passed.

Dr. J. S. Cassidy, of Covington, Ky., read a paper on "Chemistry," which occupied thirty minutes.

The Association then adjourned until Thursday morning.

THURSDAY, AUGUST 9TH, 1877.

The special order was taken up,—the report of the special committee recommending the division of the Association into permanent sections. After considerable discussion, the report was recommitted.

The next thing in order was the reading by Dr. Taft of a paper prepared by Dr. C. G. Essig, of the Committee on Chemistry, on the subject of "*Mercury and its Compounds in Dentistry.*"

The report went on to say that the objection had been brought against the red vulcanized-rubber plates that they contained free mercury, and were, therefore, poisonous. The report took the precisely opposite ground, the author detailing several tests made which showed that the red plates do not contain free mercury.

Dr. Palmer, of New York, claimed that there was free mercury in such plates, and mentioned instances where persons had suffered from using them.

Dr. Kennicott at this point endeavored to join in the discussion, but the Treasurer objected on the ground that as the gentleman had not paid his annual dues he was not entitled to the privileges of the floor.

Dr. Kennicott replied that he had tendered his dues and the Treasurer wouldn't receive them.

The Treasurer retorted, that the gentleman wasn't a delegate, until he had paid his dues, and referred to the Constitution of the Association.

Dr. Atkinson moved to allow Dr. Kennicott the privileges of the floor, but the motion was voted down.

The discussion of the subject being resumed, Dr. Kulp remarked, in reply to Dr. Palmer, that the facilities for experiment were present, and he would like to see a proof of his statements. For some reason or other the offer was not taken.

Drs. Gardner, Robertson, Noel, Osmond, Frederick and others continued the discussion, some holding that the mercury in the vulcanite was insoluble, and therefore harmless, while others took the opposite view. Each side related the results of experiments and tried to prove the correctness of its position. Dr. Judd's remarks were rather practical and were received with considerable favor. He said these experiments were likely to run, and had run, into ridiculous extremes. It was claimed that this sulphuret of mercury was insoluble. The truth was that it was just about as insoluble as arsenic, and if anybody wanted to experiment with arsenic, there was no objection to his taking a spoonful into the mouth and seeing whether it would produce any effect or not.

Dr. Shepard read the paper of Dr. C. A. Brackett, of the Committee on Therapeutics, on "*Dental Therapeutics.*"

The report considered the usefulness of the various pain-destroyers in use, spoke a good word in this connection for carbolic acid with arsenious acid, and for salicylic acid, but closed with the statement that of late years there was a growing disposition to avoid the use of any of these agents except in the very severe operations.

At this point the Association adjourned until afternoon.

On reassembling in the afternoon, Dr. F. M. Odell, of New York, read an exhaustive paper on the general subject of *Therapeutics.* The report concluded with the declaration that habits of cleanliness, good ventilation, abundant supply of good food, proper regulations as to time and quantity in regard to eating, a full satisfaction of the system in its demand for sleep, attention to business which should leave no time for sickness, were measures both prophylactic and therapeutic, and which, if followed out, would lead to the greatest earthly happiness.

Dr. Atkinson, one of the oldsst members of the Association, spoke of Dr. Odell's paper as the best correlation and statement of the nature of disease and its treatment he had ever heard. In fact, it was such an able presentation of the whole matter that he had not a single criticism to make.

Some further discussion ensued on the subjects treated of in the papers read at the morning session. Dr. Judd, Dr. Atkinson, Dr. Rehwinkel, and others had "views" to pronounce.

Dr. G. H. Cushing, of this city, Chairman of the Committee on *Operative Dentistry*, read a report on the present status of that branch of the profession. He spoke of the materials in use in the filling of teeth, and stated, as the result of an extensive correspondence with leading dentists, that the use of tin in combination with gold had been resorted to to a very limited extent, and that the use of amalgam, as well as non-cohesive gold, had largely increased and was still increasing. Operative dentistry to-day occupied a conservative position, running neither to one extreme nor the other, as regards methods. In conclusion, the report stated that one of the great needs of the present day was the organization of one or two institutions liberally endowed and independent of the tuition of pupils.

The last sentence particularly was received with applause.

A. W. Harlan, of this city, read a supplemental report on the same subject. According to this document, amalgam was far inferior to gold in fillings.

The report was briefly discussed by Dr. Rawls, Dr. Howe, Dr. Webb, and others. At 5 o'clock the Convention adjourned until evening, and the members in the meantime enjoyed a carriage-ride to Lincoln Park, up and down the Lake-Shore Drive, and back down town through the avenues on the North Side.

### Electing Officers.

The Association was called to order at 8:30 o'clock.

Dr. Keeley, of Ohio, introduced a resolution providing for amending the constitution so that in the future local Associations should be entitled to one delegate to the General Association for every fifteen members. Laid over under the rules.

On motion an order was drawn for the salaries of the Secretary and Treasurer.

Prof. Taft, of Cincinnati, introduced a resolution providing for the appointment of a committee of three to revise the nomenclature and terminology used by the profession, to report at the next meeting.

After some discussion, the resolution was adopted, and Drs. Taft, Judd, and Atkinson were appointed as the Committee.

The election of officers for the ensuing year was then proceeded with, resulting as follows:

*President.*—Dr. F. H. Rehwinkel, of Ohio.

*First Vice-President.*—Dr. L. D. Shepard, of Boston.

*Second Vice-President.*—Dr. G. T. Barker, of Philadelphia.

*Corresponding Secretary.*—Dr. M. H. Webb, of Philadelphia.

*Recording Secretary.*—Dr. M. S. Dean, of Chicago.

*Treasurer.*—Dr. W. H. Goddard, of Louisville.

*Executive Committee.*—Drs. Homer Judd, of St. Louis; J. H. Fillebrown, of Portland; and W. H. Morgan, of Nashville.

Niagara Falls was selected as the place for the next meeting.

FRIDAY, AUGUST 10TH, 1877.

After some miscellaneous business, the following gentlemen were elected honorary members of the Association:

J. Smith Turner, M. R. C. S., London, England; Mordaunt Stevens, M. D., D. D. S., Paris, France; Charles S. Tomes, M. A., M. R. C. S., and John Tomes, M. R. C. S., London, Eng.; E. Mosgitol, M. D., Paris, and Prof. Wedl, Vienna, Austria.

Drs. J. Taft, and Atkinson, took the newspapers of the city to task for styling Dr. Goddard, Treasurer, a "watch dog," and saying that he had a gruff voice. Dr. Allport, of Chicago, offered the following resolution, which, after discussion was adopted:

*Resolved:*—That this Association fully indorses the action of its Treasurer in exacting dues in full from all members before they are allowed to take part in the proceedings, and, in so doing, he is only carrying out the law. Dr. Corydon Palmer, of N. Y., delivered a brief address on Operative Dentistry, which he illustrated by figures and diagrams upon the blackboard, and which led to a spirited discussion. A letter upon the subject of transplanting teeth, by Dr. W N. Morrison, of St. Louis, was then read by the Secretary. The Committee on "Mechanical Dentistry" then submitted their report, after which the subject of Dental Education was discussed; a report being read by Dr. Thos. Fillebrown, of Maine, on Dental Colleges. In the afternoon Dr. Shepard, of Boston, offered resolutions of thanks to the proprietors of the Grand Pacific Hotel, for furnishing Hall and Stationery free of cost, which were unanimously adopted. The following Standing Committees for the ensuiug year were appointed:

*Physiology.*—M. S. Dean, C. W. Spalding, J. H. McQuillen.

*Pathology.*—G. R. Thomas, W. H. Atkinson, D. C. Hawkhurst.

*Histology and Microscopy.*—G. V. Black, G. F. Waters, C. N. Pierce.

*Chemistry.*—T. L. Buckingham, L. G. Noel, W. A. Bronson.

*Therapeutics.*—F. M. Odell, L. C. Ingersoll, C. C. Chittenden.

*Operative Dentistry.*—H. A. Smith, L. D. Shepard, M. H. Webb.

*Mechanical Dentistry.*—A. L. Northrop, H. H. Keith, W. M. Morrison.

*Education.*—J. N. Crouse, Thomas Fillebrown, G. J. Fredericks.

*Literature.*—C. D. Cook, A. H. Brockway, W. H. Morgan.

*Etiology.*—G. T. Barker, D. W. Clancy, J. F. Marriner.

*Prize Essays.*—H. Judd, W. W. Allport, C. S. Stockton.

Dr. A. H. Brockway read an interesting report on "The Introduction of Modern Inventions in the Practice of Dentisty," after which the newly elected officers were installed, and the Association adjourned, to meet at Niagara Falls in 1878.

